# Cyclo-oxygenase-2 selective inhibitors and nonsteroidal anti-inflammatory drugs: balancing gastrointestinal and cardiovascular risk

**DOI:** 10.1186/1471-2474-8-73

**Published:** 2007-08-03

**Authors:** R Andrew Moore, Sheena Derry, Henry J McQuay

**Affiliations:** 1 Pain Research and Nuffield Department of Anaesthetics, University of Oxford, The Churchill, Headington, Oxford, OX3 7LJ, UK

## Abstract

**Background:**

Differences between gastrointestinal and cardiovascular effects of traditional NSAID or cyclooxygenase-2 selective inhibitor (coxib) are affected by drug, dose, duration, outcome definition, and patient gastrointestinal and cardiovascular risk factors. We calculated the absolute risk for each effect.

**Methods:**

We sought studies with large amounts of information to calculate annualised rates for clearly defined gastrointestinal (complicated upper gastrointestinal perforations, ulcers, or bleeds, but not symptomatic or endoscopic ulcers) and serious cardiovascular outcomes (antiplatelet trial collaborators – APTC – outcome of fatal or nonfatal myocardial infarction or stroke, or vascular death).

**Results:**

Meta-analyses and large randomised trials specifically analysing serious gastrointestinal bleeding or cardiovascular events occurring with five different coxibs had appropriate data. In total there were 439 complicated upper gastrointestinal events in 49,006 patient years of exposure and 948 serious cardiovascular events in 99,400 patient years of exposure. Complicated gastrointestinal events occurred less frequently with coxibs than NSAIDs; serious cardiovascular events occurred at approximately equal rates. For each coxib, the reduction in complicated upper gastrointestinal events was numerically greater than any increase in APTC events. In the overall comparison, for every 1000 patients treated for a year with coxib rather than NSAID, there would be eight fewer complicated upper gastrointestinal events, but one more fatal or nonfatal heart attack or stroke. Three coxib-NSAID comparisons had sufficient numbers of events for individual comparisons. For every 1000 patients treated for a year with celecoxib rather than an NSAID there would be 12 fewer upper gastrointestinal complications, and two fewer fatal or nonfatal heart attacks or strokes. For rofecoxib there would be six fewer upper gastrointestinal complications, but three more fatal or nonfatal heart attacks or strokes. For lumiracoxib there would be eight fewer upper gastrointestinal complications, but one more fatal or nonfatal heart attack or stroke.

**Conclusion:**

Calculating annualised event rates for gastrointestinal and cardiovascular harm shows that while complicated gastrointestinal events occur more frequently with NSAIDs than coxibs, serious cardiovascular events occur at approximately equal rates. For each coxib, the reduction in complicated upper gastrointestinal events was numerically greater than any increase in APTC events.

## Background

Chronic pain, defined as pain of at least moderate severity, and present every day or almost every day for at least six months, affects one adult in five in Europe [1], and has a profound negative impact on quality of life [2]. NSAIDs (traditional non-selective non-steroidal anti-inflammatory drugs) and coxibs (cyclooxygenase-2 selective inhibitors) are effective analgesics and anti-inflammatory drugs, and an important pharmacological approach to pain relief, particularly chronic musculoskeletal pain. Other analgesics are available, but paracetamol in large, valid trials in osteoarthritis is no more effective than placebo [3], and opioids, alone or in combination with paracetamol, have high levels of common adverse events [4].

NSAIDs (and aspirin) are associated with upper [5] and lower [6-8] gastrointestinal harm, acute renal failure [9,10] and congestive heart failure [11,12]. Coxibs are differentiated by lower rates of upper [13-15] and lower [8] gastrointestinal harm, including endoscopic ulceration and frank bleeding events, although the only coxib currently marketed in the US now carries a black box warning for gastrointestinal complications, as do all prescription NSAIDs.

All of these drugs (aspirin, NSAIDs, and coxibs) may also be associated with increased risk of cardiovascular harm. There appears to be a dose-related effect of aspirin causing myocardial infarction in a randomised trial of patients undergoing endarterectomy [16] and in patients with colorectal polyps [17], and of coxibs in colorectal polyp trials [18] where the annual event rate with placebo was less than 0.5%. In dementia patients with an annual risk of over 2% with placebo, coxibs were not associated with more thrombotic vascular events than placebo over several years of treatment [18]. In arthritis, the annual risk with placebo is intermediate between these two conditions, at almost 1%. Increased cardiovascular effects for coxibs compared with placebo but not NSAIDs have been seen in studies in patients with arthritis [18-20]. Observational studies indicate that while some cyclooxygenase inhibitors (selective and non-selective), including aspirin, have increased risk of cardiovascular adverse events, others do not [21].

Serious gastrointestinal or cardiovascular events may be rare, but they important because they may not be reversible and can be life-threatening. There are a number of issues that complicate interpretation of available evidence and treatment decisions:

1. Demonstrating statistical significance of differences between treatment groups when events are rare requires large numbers of patients. The number of events recorded in clinical trials or observational studies is often small. Event rates are of the order of 1% a year or less, and trials frequently shorter than a year.

2. Cardiovascular events are rarely a primary outcome of trials, and so even randomised trials become, in effect, high-quality observational studies. Exceptions are the recent TARGET [22] and MEDAL [23] trials and the ongoing PRECISION study.

3. In arthritis, placebo-controlled trials may be limited to 6–12 weeks, while active controlled comparisons with NSAID can last for a year or more. The overall cumulative rate of events will vary with the duration of the trials, as will the absolute number of events.

4. Typically in RCTs and observational studies people take coxibs for longer than they take NSAIDs [24], so that there is greater exposure to coxibs than NSAIDs, even with the same number of patients in each treatment group. Analysis using crude events may be different from that using years of exposure.

5. Trials may involve different patient groups with different levels of gastrointestinal or cardiovascular risk. For example, trials have been conducted in patients with osteoarthritis, rheumatoid arthritis, and back pain, the obvious targets for anti-inflammatory analgesics, in whom extrapolated 10-year risk of an APTC (Antiplatelet Trial Collaborators [25]) event (fatal or nonfatal heart attack or stroke, or cardiovascular death) was about 10% [18]. Because inhibition of cyclooxygenase-2 enzyme has been considered to be helpful in other conditions, coxibs have also been used in patients with Alzheimer's disease (extrapolated 10-year risk of an APTC event about 22% [18]), and following colorectal cancer surgery to prevent recurrence (extrapolated 10-year risk of an APTC event about 5% [18]).

6. Some comparisons are with placebo. Others are with NSAIDs, but some NSAIDs, like naproxen, are theoretically capable of themselves reducing cardiovascular events (much like aspirin). Comparisons with naproxen and non-naproxen NSAIDs have led to much sub-group analysis, with the attendant problems [26].

7. Individual trials may use different definitions of an "event" and may report either events as reported by investigators, or events determined by an independent adjudication committee. This can make comparisons between trials, or combining trial results, difficult, because like may not always be compared with like.

8. Patients in trials may or may not be permitted to use low dose aspirin, because aspirin, even at low doses, can cause gastrointestinal problems and confound results. The exclusion of patients using aspirin may lead to selection of patients at lower cardiovascular risk than people in the community with arthritis or chronic pain who would be candidates for treatment with NSAID or coxib.

9. Clinical trials of coxibs designed to demonstrate gastrointestinal safety, have used doses of coxibs higher than the maximum licensed daily dose [13-15] for arthritis. Comparator NSAIDs have been used at the maximum daily dose. For all drugs adverse events are likely to be dose related, with higher event rates at higher doses.

Our aim was to use evidence from meta-analyses of clinical trials and cohort studies regarding gastrointestinal bleeding and APTC events to determine absolute annualised event rates for coxibs and NSAIDs, in order to clarify comparisons between them, and facilitate treatment choices.

## Methods

We looked for information on complicated gastrointestinal or serious cardiovascular outcomes in randomised controlled trials and meta-analyses of randomised trials of coxibs and NSAIDs, and in observational studies. The main gastrointestinal outcome of interest was adjudicated report of serious or complicated upper gastrointestinal perforations, ulcers, or bleeds, but not including symptomatic ulcers or ulcers detected endoscopically. Typically in randomised trials or meta-analyses of randomised trials this would involve presentation with haematemesis or melaena, perforation or obstruction, reduction in haemoglobin of 20 g/L or more, and to have ulcer or erosion in oesophagus, stomach, or duodenum. The main cardiovascular outcome of interest was the APTC outcome of fatal or nonfatal myocardial infarction or stroke, or vascular death. Other outcomes, especially myocardial infarction and stroke, were tabulated separately, together with all-cause mortality and cardiovascular mortality. Where available, adjudicated outcomes were preferred. It was anticipated that less well documented outcomes would be available in cohort studies than in randomised trials, though in practice cohort studies were not used in any analysis.

We searched PubMed (to November 2006), using individual drug names and relevant text words and indexing terms for drugs, outcomes, and study designs. We also searched bibliographies and contacted experts and companies for any additional information about available studies.

For the purposes of this analysis, information was required from patients predominantly with chronic pain (osteoarthritis, rheumatoid arthritis, back pain, ankylosing spondylitis), in studies typically of duration longer than six weeks, with coxib, NSAID or placebo treatment groups. Randomised trials (alone or in meta-analyses) needed to be double blind, and have high quality scores. Observational studies needed to be large and comprehensive.

The information required included the description and number of events, the number of patients studied, dose of drug, and the mean duration of exposure per patient, or total patient years of exposure, or data to make those calculations. Information was extracted by RAM and checked by SD.

For each coxib and NSAID comparison we calculated the annualised rate for each outcome. This was done by dividing the actual number of events observed by the number of person years of observation for each individual treatment arm, as provided in a report, and expressing the result as the number of events per 100 or per 1000 person years. Both are used because while the former is equivalent to a percentage rate per annum, it frequently results in fractions that are less easily understood for description of risk. For each coxib we calculated the difference between NSAIDs and coxibs in the number of events for 1000 patients, and expressed the result numerically, as a frequency, and in words. An assumption of constant risk over time is justified by linear risks for gastrointestinal [13,15] and cardiovascular [27] events. Where information was available in several studies (for instance in both a definitive randomised trial and a meta-analysis), we chose that with the largest number of events, or combined them, as appropriate.

## Results

Additional files contain information, including definition of outcomes, on major studies (randomised trials, meta-analyses of randomised trials, and observational studies) that were examined closely for information on annual gastrointestinal or cardiovascular event rates, or information to calculate annualised event rates [see Additional files 1 and 2]. Some studies contained information from the same, or some of the same trials, and our strategy was to use only the largest or most recent of them. Absolute risk data were available only from RCTs and meta-analyses of RCTs, and a few cohort studies; the large number of case-control studies did not have absolute risk rates so could not be used.

For complicated gastrointestinal events we chose to use data only from meta-analyses of randomised trials for celecoxib [28], etoricoxib [29], and valdecoxib [30], for rofecoxib we combined a meta-analysis [31] with a large randomised trial [13], and for lumiracoxib we chose a large randomised trial [15]. These outcomes were all adjudicated, apart from those for celecoxib [28]. All of these sources tested placebo, NSAIDs, and coxibs without resort to gastroprotective agents like histamine antagonists or proton pump inhibitors. By contrast, MEDAL [23] ensured that patients with at least one gastrointestinal risk factor received gastroprotection with a proton pump inhibitor; gastrointestinal events from this trial are considered separately.

For cardiovascular events we chose meta-analyses of randomised trials for celecoxib [19], lumiracoxib [20], rofecoxib [32], and valdecoxib [33], and for etoricoxib we combined a large randomised trial [23] with data from a meta-analysis of previous trials [34]. All outcomes were adjudicated. Low dose aspirin was not used in many trials, but was about 20% in CLASS, 22% in TARGET, and 35% in MEDAL, which formed a large part of the data set to be used. Because low dose aspirin use was a potential confounder, and because myocardial infarction was the major cardiovascular event, we performed an analysis according to use or non-use of low dose aspirin, both by events based on number of patients and by patient years of exposure, and compared both with all NSAIDs and non-naproxen NSAIDs [see Additional file 3]. These multiple comparisons failed to show any large difference in event rates between coxibs and NSAIDs according to low dose aspirin use, using more data but with a similar result to other meta-analyses [18]. We therefore used data for all patients.

For each coxib, information on complicated gastrointestinal and serious cardiovascular adverse events were well defined. For each coxib analyses of gastrointestinal and cardiovascular adverse events involved largely the same trials, and predominantly concerned patients with osteoarthritis and rheumatoid arthritis.

**Figure 1:**
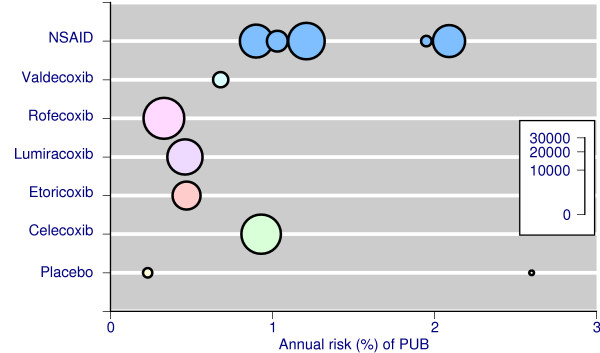
Incidence of complicated upper gastrointestinal complications in individual RCTs and meta-analyses. The size of the symbol is proportional to patient numbers (inset scale)

Table 1 and Figure 1 show the annualised rates of complicated upper gastrointestinal events (usually defined as complicated perforations, ulcers, or bleeds) for placebo, individual coxibs, and NSAIDs aggregated together. The total number of events was small with placebo (4 events in 550 patient years, Table 1), larger with coxibs (158 events in 28,000 patient years), and largest with NSAIDs (277 events in 20,400 patient years). Annualised rates were numerically higher for NSAIDs than coxibs (Table 1, Figure 1). It was not possible to analyse individual NSAIDs separately, or different doses of coxibs or NSAIDs, due to small numbers of events. Patient characteristics and baseline risks of these studies were similar. Combining all coxib studies there were, in total, 439 complicated upper gastrointestinal events in 49,000 patient years of exposure, giving an overall rate of one per 112 patients per year of exposure.

**Table 1:** Summary of available best evidence on serious upper gastrointestinal complications

**Treatment**	**Best evidence**	**Patient years of exposure**	**Number of events**	**PUB(% per year)**
Placebo	Meta-analysis of rofecoxib trials [31]	112	3	2.68
Placebo	Meta-analysis of celecoxib trials [28]	441	1	0.23

Celecoxib	Meta-analysis of celecoxib trials [28]	7943	74	0.93
Etoricoxib	Meta-analysis of etoricoxib trials [29]	4007	19	0.47
Lumiracoxib	RCT [15]	6368	29	0.46
Rofecoxib	Meta-analysis of rofecoxib trials [31,13]	8524	28	0.33
Valdecoxib	Meta-analysis of valdecoxib trials [30]	1183	8	0.68

NSAID	Meta-analysis of celecoxib trials [28]	5258	110	2.09
NSAID	Meta-analysis of etoricoxib trials [29]	2230	23	1.03
NSAID	RCT with lumiracoxib [15]	6845	83	1.21
NSAID	Meta-analysis of rofecoxib trials [31,13]	5532	50	0.90
NSAID	Meta-analysis of valdecoxib trials [30]	563	11	1.95

PUB – perforations, ulcers, bleeds; RCT – randomised controlled trial

Two cohort studies [see Additional file 1] reported information allowing calculation of annualized event rates of hospital admission with upper gastrointestinal bleed. In one [35] the annualised event rate was 0.4% for celecoxib (8,800 patient years), 0.7% for rofecoxib (5,900 patient years), and 1.3% for NSAIDs (1,400 patient years). The other, in the UK [36], had lower rates of 0.25% for coxibs (1,600 patient years) and 0.46% for NSAIDs (based on 635,000 patient years, but over decades). In general these cohort studies are in agreement with the results of meta-analyses of randomised trials (Table 1). The several case-control studies had no relevant information for annualised rates.

**Figure 2:**
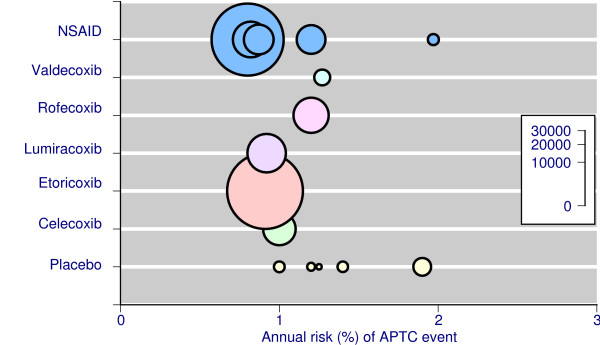
Incidence of serious cardiovascular events in individual RCTs and meta-analyses The size of the symbol is proportional to patient numbers (inset scale).

Table 2 and Figure 2 show the annualised rates of cardiovascular events (usually defined as the APTC outcome of fatal or nonfatal heart attack or stroke, or vascular death; "APTC events") for placebo, individual coxibs, and NSAIDs aggregated together. The total number of events was moderate with placebo (52 events in 3,400 patient years), larger with NSAIDs (395 events in 44,200 patient years), and largest with coxibs (501 events in 51,800 patient years). Annualised rates were similar for coxibs and NSAIDs (Table 2, Figure 2). Combining all coxib studies there were, in total, 948 cardiovascular events in 99,400 patient years of exposure, giving an overall rate of one per 105 patients per year of exposure.

**Table 2:** Summary of available best evidence on APTC events

**Treatment**	**Best evidence source [reference]**	**Patient years of exposure**	**Number of events**	**APTC(% per year)**
Placebo	Meta-analysis of celecoxib trials [19]	585	8	1.40
Placebo	Meta-analysis of etoricoxib trials [34]	335	4	1.20
Placebo	Meta-analysis of lumiracoxib trials [20]	614	6	1.00
Placebo	Meta-analysis of rofecoxib trials [32]	1678	32	1.90
Placebo	Meta-analysis of valdecoxib trials [33]	161	2	1.25

Celecoxib	Meta-analysis of celecoxib trials [19]	5651	57	1.10
Etoricoxib	Meta-analysis of etoricoxib trials [23, 34]	30404	277	0.91
Lumiracoxib	Meta-analysis of lumiracoxib trials [20]	7859	72	0.92
Rofecoxib	Meta-analysis of rofecoxib trials [32]	6556	78	1.19
Valdecoxib	Meta-analysis of valdecoxib trials [33]	1340	17	1.27

NSAID	Meta-analysis of celecoxib trials [19]	4386	54	1.20
NSAID	Meta-analysis of etoricoxib trials [23, 34]	27644	232	0.80
NSAID	Meta-analysis of lumiracoxib trials [20]	6805	55	0.82
NSAID	Meta-analysis of rofecoxib trials [32]	4726	41	0.87
NSAID	Meta-analysis of valdecoxib trials [33]	656	13	1.97

APTC – Antiplatelet Triallists Collaboration outcome of fatal and nonfatal heart attack or stroke, and cardiovascular death

Two cohort studies [see Additional file 2] reported information allowing calculation of annualised rates for comparison with meta-analyses of randomised trials. One [37] used APTC events and reported rates between 1.2% and 1.4% for controls, NSAIDs, and coxibs, with between 5,600 and 240,000 patient years of observation for each drug. The other reported hospital admission for myocardial infarction [38] with reported rates of 1.2% for celecoxib (7,000 patient years), rofecoxib (4,800 patient years), and NSAIDs (12,600 patient years). Again, these cohort studies are in agreement with the results of meta-analyses of randomised trials (Table 2).

### Gastrointestinal versus cardiovascular outcomes for individual coxib-NSAID comparisons

**Figure 3:**
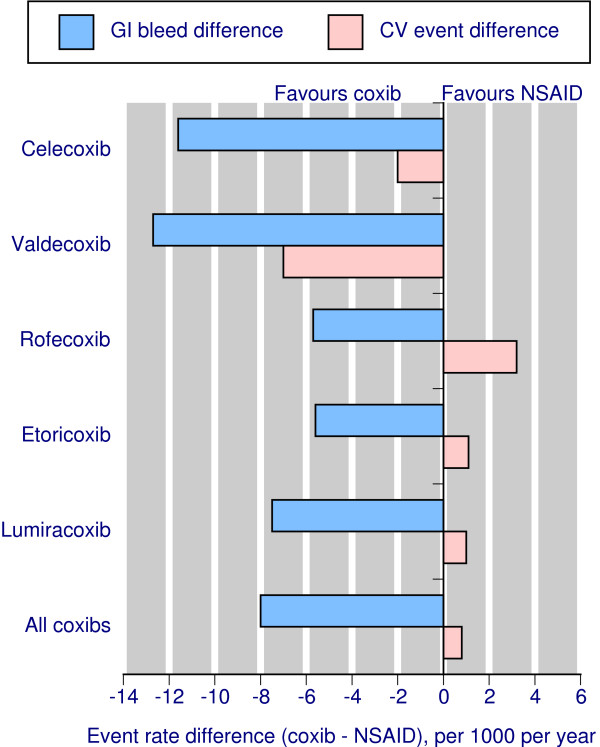
Event rates for serious gastrointestinal and cardiovascular events for each coxib, and for all coxibs combined, for 1000 patients treated for one year with each drug.

The calculations for all coxibs combined versus all NSAIDs, and for individual coxib versus all NSAIDs within direct comparisons, using the best evidence available from meta-analyses of randomised trials, are shown in Table 3, and Figure 3 where similar molecular structures are adjacent. Table 3 and Figure 3 do not consider that in all cases there was a statistically significant reduction in complicated gastrointestinal events, but no significant difference in cardiovascular events. The calculations are based solely on the absolute event rates for the two different events, gastrointestinal and cardiovascular. Calculations for all coxibs vs all NSAIDs use average annual event rates.

**Table 3:** Summary of calculations fort individual coxibs, and all coxibs combined

	Annual event rate per 100	Annual event rate per 1000	Absolute risk frequency per year		Annual event rate per 100	Annual event rate per 1000	Absolute risk frequency per year
	
**All coxib vs All NSAID**				**Lumiracoxib vs NSAID**			
**PUB**				**PUB**			
NSAID	1.36	13.6	74	NSAID	1.21	12.1	83
All coxib	0.56	5.6	179	Lumiracoxib	0.46	4.6	217
	
Difference		8.0	125	Difference		7.5	133
	
**APTC**				**APTC**			
NSAID	0.89	8.9	112	NSAID	0.82	8.2	122
All coxib	0.97	9.7	103	Lumiracoxib	0.92	9.2	109
	
Difference		-0.8	-1250	Difference		-1.0	-1000
	
**Celecoxib vs NSAID**				**Valdecoxib vs NSAID**			
**PUB**				**PUB**			
NSAID	2.09	20.9	48	NSAID	1.95	19.5	51
Celecoxib	0.93	9.3	108	Valdecoxib	0.68	6.8	147
	
Difference		11.6	86	Difference		12.7	79
	
**APTC**				**APTC**			
NSAID	1.20	12.0	83	NSAID	1.97	19.7	51
Celecoxib	1.00	10	100	Valdecoxib	1.27	12.7	79
	
Difference		2.0	500	Difference		7.0	143
	
**Rofecoxib vs NSAID**				**Etoricoxib vs NSAID**			
**PUB**				**PUB**			
NSAID	0.90	9.0	111	NSAID	1.03	10.3	97
Rofecoxib	0.33	3.3	303	Etoricoxib	0.47	4.7	213
	
Difference		5.7	175	Difference		5.6	179
	
**APTC**				**APTC**			
NSAID	0.87	8.7	115	NSAID	0.80	8.0	125
Rofecoxib	1.19	11.9	84	Etoricoxib	0.91	9.1	110
	
Difference		-3.2	-313	Difference		-1.1	-909

PUB – perforations, ulcers, bleeds; APTC – Antiplatelet Triallists Collaboration outcome of fatal and nonfatal heart attack or stroke, and cardiovascular death. In these calculations, the annualised event rates have been converted for simplicity to event rates per 1000 patients, and absolute event frequency per year calculated from that. Differences between NSAID and coxibs were calculated, and the absolute risk difference frequency calculated. This is equivalent to a number needed to prevent one event if positive, and number needed to harm if negative, for one year of treatment of coxib rather than an NSAID

Table 3 gives not only the event rates, but the absolute annual frequency of each of these events occurring with NSAID or coxib. Although different reports were used to calculate gastrointestinal and cardiovascular event rates, these reports used essentially the same trials or patient populations for each coxib, so that a direct comparison between the two different events was justified. The difference in absolute event rates expressed as a frequency is equivalent to a number needed to treat to prevent an event (if a positive number), or a number needed to harm (if a negative number).

The overall comparison shows that for every 1000 patients treated for a year with any coxib rather than any NSAID there would be eight fewer upper gastrointestinal complications, but one more fatal or nonfatal heart attack or stroke. However, within the different coxib-NSAID comparisons, there are different results:

For celecoxib, for every 1000 patients treated for a year with celecoxib rather than an NSAID there would be 12 fewer upper gastrointestinal complications, and two fewer fatal or nonfatal heart attacks or strokes.

For valdecoxib, for every 1000 patients treated for a year with coxib rather than an NSAID there would be 13 fewer upper gastrointestinal complications, and seven fewer fatal or nonfatal heart attacks or strokes.

For rofecoxib, for every 1000 patients treated for a year with coxib rather than an NSAID there would be six fewer upper gastrointestinal complications, but three more fatal or nonfatal heart attacks or strokes.

For etoricoxib, for every 1000 patients treated for a year with coxib rather than an NSAID there would be six fewer upper gastrointestinal complications, but one more fatal or nonfatal heart attack or stroke.

For lumiracoxib, for every 1000 patients treated for a year with coxib rather than an NSAID there would be eight fewer upper gastrointestinal complications, but one more fatal or nonfatal heart attack or stroke.

The large MEDAL programme of 34,700 patients over 51,000 patient years of observation compared etoricoxib (plus low dose aspirin or proton pump inhibitor where indicated) with diclofenac (plus low dose aspirin or proton pump inhibitor where indicated)[23,39]. The APTC event rate was about 0.85% in both groups, and is similar in magnitude to other results for coxibs and NSAIDs (Table 2). The confirmed complicated upper gastrointestinal event rate was about 0.3% per year in both groups, and was substantially lower than other results for NSAIDs (0.9 to 2.1% per year, Table 1), and, indeed some results for coxibs (0.3 to 0.9% per year, Table 1).

## Discussion

There are many limitations with trying to analyse the balance of gastrointestinal and cardiovascular risks for coxibs and NSAIDs.

First among them is an assumption, unstated but implicit, that these are the only choices for treating pain. They are not. Other strategies exist that have more or less evidence for benefit and harm, and encompass both conventional and unconventional therapies. The case of coxib trials is, however, unusual in the large number of patients involved in randomised trials. It has been pointed out that around 200 events may be necessary to give credibility to a result [40]. The number of patients involved in trials to gather a sufficient number of events is likely to be large in the case of rare but serious adverse events; in the tens of thousands for small differences between events occurring at 1% or so [41]. For most therapies we have insufficient information to exclude the possibility that rare but serious adverse events are present (a case of absence of evidence not equating to evidence of absence), so cannot properly evaluate the balance of benefits and risks. That should not prevent our trying to look at methods in an area not well explored, and which has been explored mainly using relative risks [18] rather than absolute event rates. In this regard it is worth noting that it has been pointed out that "*All policy decision should be based on absolute measures of risk: relative risk is strictly for researchers only" *[42], so that using an absolute risk approach would seem to be sensible. It still needs to be emphasised, though, that trying to evaluate the risks and benefits of two therapies does not deny the appropriateness of others for some patients.

Secondly, it is a common experience that particular patients do particularly well on particular medicines; while medicines may be equally efficacious on average, individual responses to the medicine can be dramatically different [43-45]. Our approach here used only average data from trials, and the experience of any individual is likely to be different, and presently not calculable.

Thirdly, this analysis is limited to only two harmful outcomes, those of serious gastrointestinal or cardiovascular harm. There are others (congestive heart failure, renal impairment, anaemia) that might be included in such an analysis, as meta-analysis of clinical trial reports for adverse events has shown [28]. Moreover, gastrointestinal and cardiovascular events need not be equivalent; for each there is a spectrum of severity, which includes death.

Fourthly, while reduction in a harmful outcome is beneficial, the analysis does not consider other benefits of therapy, such as pain relief, functioning, and quality of life.

Fifthly, there is danger that rare adverse events are captured only in small numbers, with random chance producing false results. Ideally substantial numbers of events should be available [24,46,47]. Information for valdecoxib was inadequate, with only eight gastrointestinal and 17 cardiovascular events with valdecoxib (Tables 1 and 2), and similarly small numbers for NSAID comparators. For etoricoxib, 19 and 242 events respectively with etoricoxib might also be regarded as inadequate for gastrointestinal outcomes. Data from the MEDAL program [39] differed because of the use of proton pump inhibitors, the only trial to do so, and was not used. For the other coxibs, there was a reasonable, but not overwhelming, number of events for both coxib and NSAID comparators, making comparison more robust.

Sixthly, it is likely that different drugs have different levels of cardiovascular risk, irrespective of pharmacological classification. The best evidence comes from an analysis of 3.5 million patients in case-control studies, showing differences within NSAID and coxib classes [21]. Low numbers of events made it impracticable to analyse NSAID comparators individually in this study, but comparing a particular coxib with a mix of NSAIDs may give only a partial picture. In addition, either or both risks may be dose-related and there has been a tendency to use higher than licensed doses in some randomised trials.

This analysis differs from at least one previous attempt to assess the balance of gastrointestinal and cardiovascular risks of NSAIDs and coxibs [48], which was qualitative in nature. We have attempted to remove the effects of different exposures by using the number of events per 100 or per 1000 patient years as a standard, with annualised rates justified by the observation of constant gastrointestinal and cardiovascular risk in large randomised studies [13,15,27]. The level of gastrointestinal or cardiovascular risk was the same within each comparison, because information was taken from meta-analyses of randomised trials conducted on similar patients; this is important, because the suggestion is that gastrointestinal differences between coxibs and NSAIDs are greater at higher risk [49], while significant cardiovascular differences may occur mainly at lower cardiovascular risk [21,50].

Over and above these problems comes that of comparing like with like, the same drug and dose, for the same duration, in similar patients with similar risk factors. The clearest way of doing that is to compare, for each coxib, information for both outcomes derived from meta-analysis of the largest body of data from randomised trials. Measuring both outcomes in the same population, with the same drug, dose range, and duration should ensure the best comparison. Most observational studies have had a case-control design, with no data on absolute event rates, limiting their utility for this purpose. The few with cohort designs had generally similar event rates and conclusions as the meta-analyses of randomised trials, and we used them only to confirm the trial data, not for actual calculations. We were not able to compare gastrointestinal with cardiovascular event rates at particular drug doses, because this information was not generally available.

For each coxib, there was a statistically significant reduction in the number of complicated upper gastrointestinal events compared with NSAID. The use of complicated events, rather than all adjudicated perforations, ulcers, or bleeds, excludes symptomatic ulcers and includes only the most serious events. This is a conservative approach, and reduces the number of events substantially. Including less complicated gastrointestinal events would increase the balance of gastrointestinal over cardiovascular risk.

For each coxib, no significant difference was found between coxib and NSAID for APTC events, with either small reductions for celecoxib and valdecoxib, or small increases for rofecoxib and etoricoxib. This reflects the overall results for coxibs and NSAIDs, but not between coxibs and particular NSAIDs [21].

For each coxib, the reduction in complicated upper gastrointestinal events was numerically greater than any increase in APTC events. There appeared to be a difference between individual coxibs, reflecting what is seen in observational studies [21]. The large MEDAL programme [23,39] was different from other trials, in that it compared NSAID plus proton pump inhibitor when required with coxib plus plus proton pump inhibitor when required, making it more pragmatic than experimental. The important result was finding very low rates of complicated upper gastrointestinal adverse events, indicating the potential efficacy of either strategy if implemented. Implementation is the problem, though, because the evidence is that most patients with gastrointestinal risk factors do not receive protective strategies [51], and that perhaps half of patients receiving a proton pump inhibitor with an NSAID do not take it [52].

The question then is whether complicated upper gastrointestinal and APTC events are similar or different in severity. Frank upper gastrointestinal bleeding or perforation, for any cause, carries a 5%-12% risk of death, although it may be higher in an emergency, and possibly if NSAID is also involved [53-55]. A recent case series of surgery for peptic ulcers showed that half the patients were regular NSAID users, and for urgent or emergency surgery for bleeding or perforation, 10/35 patients (29%) died [56]. In APTC events there was separate documentation of cardiovascular mortality in three studies [19,22,33], where mortality was about 29%-34% (3 deaths in 10 APTC events with placebo; 38/133 with coxib; 40/117 with NSAID). In terms of mortality, the contrasting harms seem to be similar. Subjectively, of course, heart attack or stroke may seem to be more serious than gastrointestinal bleeding, but there is little objective evidence that this is the case.

## Conclusion

Where there is a sufficiency of information, absolute event rates can be used to compare benefit and harm of rare events. Using meta-analyses of randomised trials of patients with arthritis or chronic pain, and calculating annualised event rates for major gastrointestinal and cardiovascular harm, shows that while major cardiovascular events occur at approximately equal rates with NSAIDs and coxibs, major gastrointestinal events occur more frequently with NSAIDs than coxibs. For each coxib, the reduction in complicated upper gastrointestinal events was numerically greater than any increase in APTC events. Good quality observational studies are in remarkably good agreement with results from randomised trials.

## Competing interests

RAM and HJM have received lecture fees from pharmaceutical companies. The authors have received research support from charities and government sources at various times. No author has any direct stock holding in any pharmaceutical company.

## Authors' contributions

RAM was involved with the original concept, planning the study, data extraction, analysis, and preparing a manuscript; SD with data extraction, analysis, and writing; HJM with writing and advice. All authors read and approved the final manuscript.

## Pre-publication history

The pre-publication history for this paper can be accessed here:



## Supplementary Material

Additional file 1 Summary of evidence of gastrointestinal events with coxibs. Summary of evidence of gastrointestinal events with coxibs – data from individual studies Click here for file

Additional file 2 Summary of evidence of cardiovascular events with coxibs. Summary of evidence of cardiovascular events with coxibs – data from individual studies Click here for file

Additional file 3 Data and calculations for myocardial infarction with and without low dose aspirin in association with coxib and NSAID. Data and calculations for myocardial infarction with and without low dose aspirin in association with coxib and NSAID Click here for file
