# Supplementary material for: Cyclo-oxygenase-2 selective inhibitors and nonsteroidal anti-inflammatory drugs: balancing gastrointestinal and cardiovascular risk
Source: BMC Musculoskelet Disord. 2007 Aug 3;8:73. doi: 10.1186/1471-2474-8-73 (PMC2001315; doi:10.1186/1471-2474-8-73)
Supplement: Additional file 1 — Summary of evidence of gastrointestinal events with coxibs. Summary of evidence of gastrointestinal events with coxibs – data from individual studies [file 1471-2474-8-73-S1.pdf]

Additional file 1: Summary of evidence of gastrointestinal events with coxibs

Reference	Study design	Population	Main outcomes	Main results of study<br>Events reported, event rate per 100 patient years, or percentage	Relative risk (95% confidence interval)	Exposure<br>Duration, patient years of exposure, patient years per patient	Events per 100 py, number of events<br>Complicated PUB	Events per 100 py, number of events<br>PUB and symptomatic ulcer
**Large randomised trials**								
Bombardier et al. N Engl J Med 2000 343: 1520-1528	Randomised trial powered for PUB outcome, comparing 50 mg rofecoxib with 1000 mg naproxen daily	8,076 patients with RA, at least 50 years	Confirmed clinical upper GI events (perforation, bleeding symptomatic ulcer)	177 events, 53 complicated<br>Confirmed events at 2.1/100 pt years with rofecoxib, 4.5 with naproxen<br>Complicated 0.6 and 1.4 per 100 pt years	0.5 (0.3 to 0.6)<br>0.4 (0.2 to 0.8)	Median duration 9.0 months<br>R 3035 py<br>N 3022 py	R 0.6/100 py (16/4047)<br>N 1.4/100 py (37/4029)	R 2.1/100 py (56/4047)<br>N 4.5/100 py (121/4029)
Silverstein et al. JAMA 2000 284: 1247-1255	Randomised trial powered for PUB outcome, comparing 800 mg celecoxib with 2400 mg ibuprofen and 150 mg diclofenac daily	8,059 patients with OA or RA, ≥18 years	Confirmed upper GI ulcers and complication (bleeding, perforation, obstruction)	83 events including symptomatic ulcers, 35 complicated<br>Confirmed events at 2.1/100 pt years with celecoxib, 3.5 with NSAID<br>Complicated at 0.8 and 1.5 per 100 pt years	0.6 (0.4 to 0.9)<br>0.5 (0.3 to 1.1)	Duration 6 months<br>C 1441 py<br>N 1384 py	C 2.2/100 py (32/3987)<br>N 3.7/100 py (51/3981)	C 2.1/100 py (30/3987)<br>N 3.5/100 py (49/3981)
Schnitzer et al. Lancet 2004 364: 665-674	Randomised trial powered for PUB outcome, comparing 400 mg lumiracoxib with 2400 mg ibuprofen and 1000 mg naproxen daily	18,325 patients with OA, at least 50 years<br>13,506 py in safety evaluation	Confirmed upper GI ulcers and complication (bleeding, perforation, obstruction)	283 events, 112 complicated<br>Confirmed events at 1.0% with lumiracoxib, 1.5% with NSAID<br>Complicated events at 0.3% with lumiracoxib, 0.9% with NSAID	0.7 (0.5 to 0.8)<br>0.3 (0.2 to 0.5)	L 6838 py<br>N 6845 py<br>(overall average 0.75 pypt)	L 0.42/100 py (29/9117)<br>N 1.20/100 py (83/9127)	L 0.95/100 py (87/9117)<br>N 2.0/100 py (186/9127)
**Meta-analyses of randomised trials**								
Langman et al. JAMA 1999 282: 1929-1933	Prescified meta-analysis of eight randomised trials of rofecoxib versus NSAIDs	OA patients, mean age 63 years<br>n=5,435	Confirmed clinical upper GI events (perforation, bleed, ulcer)	35 confirmed complicated events<br>Complicated events at 1.3/100 pt years with rofecoxib, 2.6 with NSAID	0.5 (0.3 to 1.0)	R 1428 py (0.43 pypt)<br>N 615 py (0.39 pypt)<br>P 112 py (0.22 pypt)	R 1.3/100 py (19/3357)<br>N 1.8/100 py (16/1564)<br>P 2.6/100 py (3/514, for shorter duration studies)	
Goldstein et al. Am J Gastroenterol 2000 95: 1681-1690	Meta-analysis of 14 randomised trials of celecoxib versus NSAIDs	OA or RA patients, mean age 60 years<br>n=11,008	Confirmed clinical upper GI events (perforation, bleed, ulcer)	11 confirmed complicated events<br>Complicated events at 0.2/100 pt years with celecoxib, 1.7 with NSAID	0.2 (0.1 to 0.5)	C 1020 py (0.16 pypt)<br>N 535 py (0.19 pypt)<br>P 208 py (0.11 pypt)	C 0.2/100 py (2/6376)<br>N 1.7/100 py (9/2768)<br>P 0/100 py (0/1864)	
Edwards et al. Pain 2004 111: 286-296	Meta-analysis of nine randomised trials of valdecoxib versus NSAIDs	OA or RA patients, n=5,726	Clinically significant upper GI bleed	10 confirmed complicated events<br>Complicated event rate 0.1% with valdecoxib, 0.4% with NSAID	0.2 (0.04 to 0.8)	Mean exposure not available (6/12/26 week trials)	V 2/2733 (0.1% of patients)<br>N 8/1846 (0.4% of patients)	
Goldstein et al. Aliment Pharmacol Ther 2004 20: 527-538	Meta-analysis of eight randomised trials of valdecoxib versus NSAID	OA or RA patients, mean age 58 years<br>n=7,434	Confirmed clinical upper GI events (perforation, bleed, ulcer)	88 symptomatic ulcers, 19 complicated<br>Symptomatic + complicated 0.8% with valdecoxib, 3.3% with NSAID<br>Complicated 0.2% with valdecoxib, 0.5% with NSAID	0.3 (0.2 to 0.4)<br>0.4 (0.1 to 0.9)	V 1183 y (0.27 pypt)<br>N 563 y (0.27 pypt)	V 0.68/100 py (8/4362)<br>N 1.94/100 py (11/2099)	V 2.7/100 py (32/4362)<br>N 11.8/100 py (69/2099)

Hooper et al. BMJ 2004 329: 948	Meta-analysis of 17 randomised trials of coxibs versus NSAIDs	n=25,564	Variety of outcomes reported, including serious gastrointestinal complications, and symptomatic ulcers	114 serious gastrointestinal complications, 0.36% with coxib, 0.73% with NSAID<br>288 symptomatic ulcers, 0.8% with coxib, 1.8% with NSAID	0.5 (0.4 to 0.8)<br>0.5 (0.4 to 0.6)	Mean exposure not available		
Watson et al. Curr Med Res Opin 2004 20: 1539-1548	Meta-analysis of 20 randomised trials of rofecoxib versus NSAIDs	OA or RA patients, n=10,026 rofecoxib, 7,046 NSAID	Confirmed and investigator reported PUBs, and complicated	97 confirmed PUBs, 0.43% with rofecoxib, o.76% with NSAIDs	Confirmed<br>0.4 (0.2 to 0.5)<br>Complicated<br>0.4 (0.2 to 0.9)	R 5849 y (0.58 pypt)<br>N 2889 y (0.41 pypt)	R 0.21/100 py (12/10026)<br>N 0.45/100 py (13/7046)	
Moore et al. Arth Res Ther 2005 7: R644-R655	Meta-analysis of 31 randomised trials of celecoxib versus NSAIDs	OA or RA<br>n=39,605 (31,171 in analusis of ulcers and bleeds)	Variety of outcomes reported including clinical ulcers and bleeds	184 clinical ulcers or bleeds, 0.4% with celecoxib, 0.9% with NSAID	0.6 (0.5 to 0.8)	C 7943 py<br>N 5258 py<br>P 441 py<br>overall exposure 0.42 pypt)	C 0.9/100py (74/18756)<br>N 2.1/100 py (110/12415)<br>P 0.2/100 py (1/2859)	
Ramey et al. Curr Med Res ther 2005 21: 715-722	Meta-analysis of 10 randomised trials involving etoricoxib and NSAIDs	OA, RA, ankylosing spondylitis<br>n=5,441	Variety of outcomes reported, including all PUB and complicated PUB, both investigator reported and adjudicated	111 investigator reported PUBs<br>95 confirmed PUBs, 1.2% with etoriocoxib, 2.5% with NSAID	0.5 (0.3 to 0.7)	E 1.24 pypt<br>N 1.00 pypt	E 0.47/100 py (19/3226)<br>N 1.03/100 py (23/2215)	E 1.0/100 py (40/3226)<br>N 2.5/100 py (55/2215)
**Large observational studies**								
Mamdani et al. BMJ 2002 325: 624-630	Observational cohort study	Users of NSAID, coxib, or non users. Total population about 144,000<br>Rofecoxib 92% ≤25 mg daily; celecoxib 80% ≤200 mg daily)	Hospital admission for upper GI bleeding	82 events with controls, 17 with NSAID, 75 with coxib<br>Rofecoxib, but not celecoxib had significantly greater association with bleeding than controls	Celecoxib compared with NSAID<br>0.2 (0.1 to 0.4)<br>Rofecoxib compared with NSAID<br>0.5 (0.3 to 1.0)	Coxib 14683 py (Cele 8818 Rofe 5865)<br>N 1353<br>Community 37981	C 0.4/100 py (32/18908)<br>R 0.7/100 py (43/14583)<br>N 1.3/100 py (17/1353)<br>Community 0.2/100 py (82/100,000)	
MacDonald et al. Gut 2003 52: 1265-1270	Retrospective cohort analysis	Users of NSAID, coxibs, and non users. Total 26,000 incident cases of upper GI haemorrhage	Hospital admission for upper GI bleeding in high risk patients	2,875 events on NSAID, 4 on coxib<br>Adjusted relative risk	0.4 (0.1 to 1.0)	Control 3136000 py<br>Coxibs 1600 py<br>N 635000 py	Control 0.08/100 py (2397 events)<br>Coxibs 0.26/100 py (4 events)<br>N 0.46/100 py (2911 events)	
Norgard et al. Aliment Pharmacol Ther 2004 19: 817-825	Population based case-control study	Users of NSAID, coxibs, and non users. 780 incident cases in patients with high risk of GI bleeding	Hospital admission for upper GI bleeding	35 patients had been exposed to coxib (4.5%)<br>97 patients had been exposed to NSAID (12%)<br>Rofecoxib, but not celecoxib had significantly greater association with bleeding than controls	0.4 (0.3 to 0.5)	Not applicable		

Abbreviations: PUB - perforations, ulcers, bleeds; OA - osteoarthritis; RA - rheumatoid arthritis; GI - gastrointestinal; n = number of patients; pt - patient; py - patient year; pypt - patient years per patient; R - rofecoxib; C - celecoxib; E - etoricoxib; V - valdecoxib; L - lumiracoxib; N - NSAID; P - placebo
