# Supplementary material for: Cyclo-oxygenase-2 selective inhibitors and nonsteroidal anti-inflammatory drugs: balancing gastrointestinal and cardiovascular risk
Source: BMC Musculoskelet Disord. 2007 Aug 3;8:73. doi: 10.1186/1471-2474-8-73 (PMC2001315; doi:10.1186/1471-2474-8-73)
Supplement: Additional file 2 — Summary of evidence of cardiovascular events with coxibs. Summary of evidence of cardiovascular events with coxibs – data from individual studies [file 1471-2474-8-73-S2.pdf]

Additional file 2: Summary of evidence of cardiovascular events with coxibs

Reference	Study design	Population	Main outcomes	Statistical outcome	Exposure Duration, patient years of exposure, patient years per patient	Events per 100 py, number of events, or percentage					
						Mortality	APTC endpoint (fatal and non-fatal MI and stroke, plus cardiovascular death)	MI	Non-fatal MI	Stroke	Non-fatal Stroke
**Large randomised trials**											
Bombardier et al. N Engl J Med 2000 343: 1520-1528	Randomised trial powered for PUB outcome, comparing 50 mg rofecoxib with 1000 mg naproxen daily	8,076 patients with RA, at least 50 years		Significant increased risk of MI with rofecoxib	Median duration 9.0 months<br>R 3035 py<br>N 3022 py	R 0.67/100 py (0.5%)<br>N 0.53/100 py (0.4%)<br>Cadiovascular death<br>R 0.27/100 py (0.2%)<br>N 0.27/100 py (0.2%)		R 0.53/100 py (0.4%)<br>N 0.13/100 py (0.1%)		R 0.27/100 py (0.2%)<br>N 0.27/100 py (0.2%)	
Silverstein et al. JAMA 2000 284: 1247-1255	Randomised trial powered for PUB outcome, comparing 800 mg celecoxib with 2400 mg ibuprofen and 150 mg diclofenac daily	8,059 patients with OA or RA, ≥18 years		No significant difference	Duration 6 months<br>C 1441 py<br>N 1384 py			C 0.50/100 py (10/3987)<br>N 0.56/100 py (11/3981)		C 0.26/100 py (5/3987)<br>N 0.5/100 py (10/3981)	
Farkouh et al. Lancet 2004 364: 675-684	Randomised trial powered for PUB outcome, comparing 400 mg lumiracoxib with 2400 mg ibuprofen and 1000 mg naproxen daily	18,325 patients with OA, at least 50 years<br>13,506 py in safety evaluation		No significant difference	52 weeks<br>(0.75 pypt)	L 23/9156 (0.25%)<br>N 22/9169 (0.24%)<br>Cardiovascular death<br>L 19/9156 (0.21%)<br>N 18/9169 (0.20%)	L 1.15/100 py (59/9156)<br>N 1.0/100 py (50/9169)	L 23/9156 (0.25%)<br>N 17/9169 (0.19%)	L 18/9156 (0.20%)<br>N 9/9169 (0.10%)	L 24/9156 (0.25%)<br>N 23/9169 (0.24%)	L 20/9156 (0.22%)<br>N 20/9169 (0.22%)
Cannon et al. Lancet 2006 online publication Nov 13: DOI:10.1016/S0140-6736(06)69666-9	Prespecified pooled analysis of three randomised trials powered for cardiovascular outcomes comparing etoricoxib 60 mg or 90 mg with diclofenac 150 mg daily	34,701 patients with OA or RA followed for an average of 18 months and >50,000 patient years of observation	Thrombotic events, Arterial thrombotic events, APTC events	No significant difference	Median duration 19 months<br>E 25836<br>D 24766	Cardiovascular deaths<br>E 37/25836 (1.3%)<br>D 26/24766 (1.0%)	E 231/26402 (0.87%)<br>D 232/25416 (0.91%)	E 111/26402 (0.42%)<br>D 122/25416 (0.48%)	E 105/26402 (0.40%)<br>D 105/25416 (0.41%)	E 89/26402 (0.33%)<br>D 79/25416 (0.31%)	E 83/26402 (0.31%)<br>D 77/25416 (0.29%)
**Meta-analyses of randomised trials**											
Matchaba et al. Clin Ther 2005 27: 1196-1214	Meta-analysis of all lumiracoxib studies in arthritis longer than 1 week	22 RCTs in arthritis with 34,668 patients, 22,781 in trials of one year. Mean age 62 years, over 50% hypertension at baseline, 6% diabetes	Lumiracoxib doses 100-800 mg daily APTC endpoint	No signirficant differences by type of comparator, duration, prospective adjudication, or dose of lumiracoxib	0.19 pypt placebo trials<br>L 1332 py<br>P 614 py<br>0.65 pypt active trials<br>L 7859 py<br>N 6805 py		L 1.1/100 py (15/7011)<br>P 1.0/100 py (6/3234)<br>L 0.92/100 py (72/12090)<br>N 0.82/100 py (55/10469)	L 0.45/100 py (6/7011)<br>P 0.33/100 py (2/3234)<br>L 0.37/100 py (29/12090)<br>N 0.29/100 py (20/10469)		L 0.30/100 py (4/7011)<br>P 0.49/100 py (3/3234)<br>L 0.37/100 py (29/12090)<br>N 0.31/100 py (21/10469)	
Simon et al. Arthritis Rheum 2005 52: S406	Meta-analysis of all celecoxib studies of 2 weeks to 3 years	41 RCTS, mainly in arthritis but including AS, back pain, and Alzheimer's disease<br>44,308 patients, 24,993 on celecoxib, 4,057 on placebo, 13,990 on NSAID	APTC endpoint, with main analysis for ≥200 mg celecoxib daily	No signirficant differences by type of comparator, except for stroke, where a significantly lower event rate was found for celecoxib compared with NSAIDs	Placebo comparator<br>P 585 py (0.14 pypt)<br>C 1268 py (0.17 pypt)<br>NSAID comparator<br>C 5651 (0.29 pypt)<br>N 4386 (0.31 pypt)	P 1.0/100 py (6/4025)<br>C 1.3/100 py (17/7462)<br>Cardiovascular death<br>P 0.51/100 py (3/4025)<br>C 0.87/100 py (11/7462)<br>NSAID comparisons<br>C0.67/100 py (30/19773)<br>N 0.75/100 py (33/13990)<br>Cardiovascular death<br>C 0.27/100 py (15/19773)	P 1.4/100 py (8/4025)<br>C1.8/100 py (23/7462)<br>NSAID comparisons<br>C 1.0/100 py (57/19773)<br>N 1.2/100 py (54/13990)	P 0.68/100 py (4/4025)<br>C 1.3/100 py (16/7462)<br>NSAID comparisons<br>C 1.1/100 py (62/19773)<br>N 0.68/100 py (42/13990)		P 1.0/100 py (6/4025)<br>C 0.87/100 py (11/7462)<br>NSAID comparisons<br>C 0.27/100 py (15/19773)<br>N 0.75/100 py (33/13990)	
White et al. Am J Ther 2004 11: 244-250	Meta-analysis of all valdecoxib studies of at least 4 weeks	RCTs for arthritis, with 7934 patients<br>4531 on valdecoxib, 1142 on placebo, 2261 on NSAIDs<br>Mean age about 70 years, with 45% history of CV disease	Doses of valdecoxib 10 to 80 mg daily	No significant difference by dose, or by duration	Placebo 161 py (0.14 pypt)<br>Valdecoxib 1340 py (0.30 pypt)<br>NSAID 656 py (0.29 pypt)	P 0.00/100 py (0/1142)<br>V 0.51/100 py (7/4531)<br>N 0.76/100 py (5/2261)<br>Cardiovascular deaths<br>P 0.00/100 py (0/1142)<br>V 0.30/100 py (4/4531)<br>N 0.45/100 py (3/2261)	Serious CV thrombotic event<br>P 1.25/100 py (2/1142)<br>V 1.27/100 py (17/4531)<br>N 1.97/100 py (13/2261)	P 0.46/100 py (1/1142)<br>V 0.43/100 py (6/4531)<br>N 1.07/100 py (7/2261)		P 0.46/100 py (1/1142)<br>V 0.60/100 py (8/4531)<br>N 0.76/100 py (5/2261)	

Konstam et al. Circulation 2001 104: 2280-2288	Meta-analysis of rofecoxib studies of at least four weeks, with longest duration 4 years	RCTs on arthritis, back pain, and Alzheimer's disease<br>Mean age 54 to 73 years in study groups, about half with one coronary risk factor	Doses of rofecoxib 12.5 to 50 mg<br>APTC endpoint	No significant difference by condition, or comparator (excepting, just, naproxen), with possible dose-response	Placebo controlled<br>R 2189 py (0.35 pypt)<br>P 1678 py (0.48 pypt)<br>NSAID controlled<br>R 6556 py (0.48 pypt)<br>N 4726 py (0.44 pypt)		Placebo controlled<br>R 1.5/100 py (33/6290)<br>P 1.9/100 py (32/3482)<br>NSAID controlled<br>R 1.2/100 py (78/13632)<br>N 0.87/100 py (41/10625)	Placebo controlled<br>R 0.86/100 py (19/6290)<br>P 0.77/100 py (13/3482)<br>NSAID controlled<br>R 0.78/100 py (51/13632)<br>N 0.45/100 py (21/10625)		Placebo controlled<br>R 0.36/100 py (8/6290)<br>P 0.90/100 py (15/3482)<br>NSAID controlled<br>R 0.38/100 py (25/13632)<br>N 0.41/100 py (19/10625)
Curtis et al. Arthritis Rheum 2003 48(suppl 9): 1600	Meta-analysis of etoricoxib studies of at least 4 weeks	Over 6,700 patients, predominantly in arthritis, ankylosing spondylitis and back pain	Does of etoricoxib ≥60 mg daily<br>Thrombotic serious adverse experiences	No significant difference between etoricoxib, placebo, or NSAIDs	Placebo controlled<br>E 560 py (0.20 pypt)<br>P 335 py (0.19 pypt)<br>NSAID controlled<br>E 4002 py (1.2 pypt)<br>N 2228 py (1.0 pypt)		Serious CV thrombotic event<br>Placebo controlled<br>E 1.3/100 py (7/2818)<br>P 1.2/100 py (4/1767)<br>NSAID controlled<br>E 1.1/100 py (46/3226)<br>N 0.8/100 py (18/2215)	E 0.35/100 py (2/2818)<br>P 0.0/100 py (0/1767)<br>NSAID controlled<br>E 0.42/100 py (17/3226)<br>N 0.31/100 py (7/2215)		E 0.53/100 py (3/2818)<br>P 0.60/100 py (2/1767)<br>NSAID controlled<br>E 0.35/100 py (14/3226)<br>N 0.18/100 py (4/2215)
Kearney et al. BMJ 2006 332: 1302-1308	Meta-analysis of all coxib vs NSAID or placebo trials of at least 4 weeks	145,373 patients, predominantly in arthritic conditions, but including colorectal polyp prevention and Altzheimer's<br>Note, large difference in polyp studies, no difference in Alkzheimer's studies)	Serious vascular events (MI, stroke, vascular death)	Overall, no significant difference between coxib and NSAID. Lower rates for naproxen	Coxib 18490 py<br>NSAID 12639 py	Vascular death:<br>Coxib 0.3/100 py (60/18490)<br>NSAID 0.2/100 py (30/12639)	Coxib 1.2/100py (216/18490)<br>NSAID 0.9/100 py (112/12639)	Coxib 0.6/100py (113/18490)<br>NSAID 0.3/100 py (42/12639)		Coxib 0.4/100py (70/18490)<br>NSAID 0.4/100 py (53/12639)
**Large observational studies**										
Mamdani et al. Arch Intern Med 2003 163: 481-486	Users of NSAID, coxib, or non users. Total population about 167,000<br>Rofecoxib 92% ≤25 mg daily; celecoxib 80% ≤200 mg daily)	Patients aged >65 years<br>Users of coxib and NSAIDs were more likely to have had hospital admission in past year, use more drugs (ACEI, aspirin, hyopglycaemic, CCB, diuretics, oestrogen, lipid lowering drugs, and nitrates), and have lower income status	Hospital admission for acute myocardial infarction	No significant difference between nonusers and users of coxibs or NSAIDs	Controls 51194 py (0.51 pypt)<br>C 7004 py (0.46 pypt)<br>R 4806 (0.40 pypt)<br>NSAID 12644 (0.32 pypt)			Control 0.82/100 py (419/100,000)<br>C 1.1/100 py (75/15271)<br>R 1.2/100 py (58/12156)<br>N 1.2/100 py (149/39537)		
Ray et al. Lancet 2002 360: 1071-1073	Users of NSAID, coxib, or non users. Total population about 378,000<br>Rofecoxib by dose ≤25 mg daily or >25 mg	Patients aged 50-84 years, not in nursing home, no history of non-CV life threatening illness<br>Users of coxibs and NSAIDs were to have major CV diseases, with treatment in last year, be taking a CV drug, or corticosteroids, have more prescriptions	Acute myocardial infarction and fatal coronary heart disease	Significant difference only for rofecoxib>25 mg daily	Controls 237975 py (1.18 pypt)<br>Ibu 16330 py (0.28 pypt)<br>Nap 21093 py (0.30 pypt)<br>C 5643 py (0.25 pypt)<br>R ≤25 4037 py (0.20 pypt)		Control 1.3/100 py (3085/202916)<br>Ibu 1.2/100 py (190/59007)<br>Nap 1.2/100 py (245/70384)<br>C 1.3/100 py (74/22337)<br>R 1.4/100 py (55/20245)			
Hernandez-Diaz et al. Basic Clin Pharmacol Toxicol 2006 98: 266-274	Meta-analysis of studies of users of NSAID, coxib or non-users	Patients predominantly >40 years. Most frequent indication was OA. NSAID users more likely to have CV risk factors than non-users: coxib users had more CV risk factors than NSAID or non-users. All stidies attempted to adjust for confounders	Myocardial infarction	Significant increase for rofecoxib (dose response) and diclofenac, and possibly for ibuprofen. Possible small decrease for naproxen without concommitant low dose aspirin	Total population approximately 3.5 million, with about 68,000 Mis			Feom all studies, compared with nonuse, RR for use (fixed effects) were:<br>All NSAIDs 1.1 (1.06 to 1.13)<br>Naproxen 0.98 (0.92 to 1.05)<br>Ibuprofen 1.07 (1.02 to 1.12)<br>Diclofenac 1.44 (1.32 to 1.56)<br>Celecoxib 0.96 (0.90 to 1.02)<br>Rofecoxib 1.26 (1.17 to 1.36)		

Abbreviations: RCT - randomised controlled trial; OA - osteoarthritis; RA - rheumatoid arthritis; CV - cardiovascular; ACEI - angiotensin converting enzyme inhibitor; CCB - calcium channel blocker; APTC - antiplatelet triallist collaboration; n = number of patients; pt - patient; py - patient year; pypt - patient years per patient; R - rofecoxib; C - celecoxib; E - etoricoxib; V - valdecoxib; L - lumiracoxib; N - NSAID; P - placebo; Ibu - ibuprofen; Nap - naproxen
