# Supplementary material for: Cyclo-oxygenase-2 selective inhibitors and nonsteroidal anti-inflammatory drugs: balancing gastrointestinal and cardiovascular risk
Source: BMC Musculoskelet Disord. 2007 Aug 3;8:73. doi: 10.1186/1471-2474-8-73 (PMC2001315; doi:10.1186/1471-2474-8-73)
Supplement: Additional file 3 — Data and calculations for myocardial infarction with and without low dose aspirin in association with coxib and NSAID. Data and calculations for myocardial infarction with and without low dose aspirin in association with coxib and NSAID [file 1471-2474-8-73-S3.pdf]

Additional file 3: Data and calculations for myocardial infarction with and without low dose aspirin in association with coxib and NSAID

Data

Trial	Coxib	NSAID	Number of Mis	Number of patients	Patient years of exposure	Number of Mis	Number of patients	Patient years of exposure	Average years on treatment	
**All patients**										
			**Coxib**			**NSAID**			**Coxib**	**NSAID**
Class	Celecoxib	Ibuprofen	7	1997	722	7	1985	690	0.36	0.35
Class	Celecoxib	Diclofenac	11	1990	719	4	1996	694	0.36	0.35
Vigor	Rofecoxib	Naproxen	20	4047	3035	4	4029	3022	0.75	0.75
Target	Lumiracoxib	Ibuprofen	5	4376	3326	7	4397	3034	0.76	0.69
Target	Lumiracoxib	Naproxen	18	4741	3651	10	4730	3595	0.77	0.76
Medal	Etoricoxib	Diclofenac	111	16833	25858	122	16504	24797	1.54	1.50
**Patients not taking low dose aspirin**										
			**Coxib**			**NSAID**			**Coxib**	**NSAID**
Class	Celecoxib	Ibuprofen	6	1574	569	2	1567	545	0.36	0.35
Class	Celecoxib	Diclofenac	7	1580	571	2	1602	557	0.36	0.35
Vigor	Rofecoxib	Naproxen	20	4047	3035	4	4029	3022	0.75	0.75
Target	Lumiracoxib	Ibuprofen	4	3401	2585	5	3431	2367	0.76	0.69
Target	Lumiracoxib	Naproxen	10	3549	2733	4	3537	2688	0.77	0.76
Medal	Etoricoxib	Diclofenac	65	11104	17057	61	10918	16404	1.54	1.50
**Patients who were taking low dose aspirin**										
			**Coxib**			**NSAID**			**Coxib**	**NSAID**
Class	Celecoxib	Ibuprofen	1	423	153	5	418	145	0.36	0.35
Class	Celecoxib	Diclofenac	4	410	148	2	394	137	0.36	0.35
Vigor	Rofecoxib	Naproxen	0	0	0	0	0	0	0.75	0.75
Target	Lumiracoxib	Ibuprofen	1	975	741	2	966	667	0.76	0.69
Target	Lumiracoxib	Naproxen	8	1192	918	6	1193	907	0.77	0.76
Medal	Etoricoxib	Diclofenac	46	5729	8801	61	5586	8393	1.54	1.50
